# Stem Cell-Based Therapy for Diabetic Foot Ulcers

**DOI:** 10.3389/fcell.2022.812262

**Published:** 2022-02-01

**Authors:** Qian Yu, Guo-hong Qiao, Min Wang, Li Yu, Yaoxiang Sun, Hui Shi, Tie-liang Ma

**Affiliations:** ^1^ Department of Hepatology, Songjiang Hospital Affiliated to Nanjing Medical University, Shanghai, China; ^2^ Department of Clinical Laboratory, The Affiliated Yixing Hospital of Jiangsu University, Yixing, China; ^3^ Jiangsu Key Laboratory of Medical Science and Laboratory Medicine, Institute of Stem Cell, School of Medicine, Jiangsu University, Zhenjiang, China

**Keywords:** diabetic foot ulcers, stem cell therapy, angiogenesis, anti-inflammatory, diabetic neuropathy

## Abstract

Diabetic foot ulcer has become a worldwide clinical medical challenge as traditional treatments are not effective enough to reduce the amputation rate. Therefore, it is of great social significance to deeply study the pathogenesis and biological characteristics of the diabetic foot, explore new treatment strategies and promote their application. Stem cell-based therapy holds tremendous promise in the field of regenerative medicine, and its mechanisms include promoting angiogenesis, ameliorating neuroischemia and inflammation, and promoting collagen deposition. Studying the specific molecular mechanisms of stem cell therapy for diabetic foot has an important role and practical clinical significance in maximizing the repair properties of stem cells. In addition, effective application modalities are also crucial in order to improve the survival and viability of stem cells at the wound site. In this paper, we reviewed the specific molecular mechanisms of stem cell therapy for diabetic foot and the extended applications of stem cells in recent years, with the aim of contributing to the development of stem cell-based therapy in the repair of diabetic foot ulcers.

## Introduction

Diabetic foot is the most common complication of diabetes and is prone to recurrence and infection. If this occurs in individuals who have an impaired immune function, the wound will become a portal to infection, leading to the presence of sepsis and amputation (Heublein et al., 2015), which is highly correlated with increased mortality. 85% of lower limb amputations are caused by diabetic foot ulcers (DFUs) (Boulton, 2010). In the absence of effective interventions, many DFU patients have experienced multiple amputations throughout their lives. Compared with people without diabetes, diabetic patients have 15 times higher amputation rates of lower limbs (Nicolucci et al., 2006). Chronic ulcers are difficult to cure, prone to recurrence, and have a high rate of amputation, significantly reducing the patients’ quality of life. It is a source of psychological burden and economic pressure for patients and places a substantial financial burden on their families and the health care system in general. Therefore, there is an unmet clinical need for new approaches to accelerate diabetic wound healing, especially in comorbidities (e.g., lower extremity ischemic disease, cardiovascular disease).

The traditional treatment of diabetic foot is mainly medical treatment and surgical blood flow reconstruction. However, for foot ischemia caused by arterial stenosis and occlusion, medical treatment cannot fundamentally solve the problem. Surgical treatment as an effective method to restore blood flow reconstruction also faces some problems. For example, the lower extremity artery lesions in diabetic foot patients mostly involve the lower leg artery (Qin et al., 2016), and many patients lack the distal arterial outflow tract (Houlind, 2020). These patients often face the risk of amputation due to the inability to receive arterial bypass or interventional therapy. Furthermore, patients with diabetic foot are often accompanied by cardiovascular and cerebrovascular diseases, so they cannot withstand the irritation of distal bypass surgery.

Lately, efforts have been made to develop innovative and effective therapies to repair chronic wounds, including topical application of growth factors or cell-based therapies. Becaplermin, a recombinant platelet-derived growth factor, is the only drug approved by the FDA to treat diabetic neuropathic ulcers. It has similar biological activities to endogenous platelet-derived growth factors, including improving chemotactic recruitment of cells involved in wound repair, promoting cell proliferation as well as angiogenesis, and enhancing granulation tissue formation (Regranex, 2008). However, the use of Becaplermin is facing several problems, including low systemic bioavailability and malignant tumors far from the application site. Besides, it is not clear whether it is valid for diabetic ischemic ulcers. Therefore, although growth factors have been shown to play an essential role in DFUs, it is necessary to explore new treatments to deal with the possible adverse consequences of diabetic foot. In addition, it is now possible to enhance the delivery efficiency of growth factors through cellular therapy (Gauglitz and Jeschke, 2011).

Although both growth factors and stem cells have defects in repairing diabetic foot, compared with the direct application of growth factors, the main advantage of stem cells in diabetic foot is that stem cells can regulate tissue regeneration in an all-around way by improving the microenvironment at the wound site. The individual use of single cytokines or growth factors obviously oversimplifies the complexity of the wound healing process involved.

Stem cells are the critical cells in post-injury and routine homeostasis skin repair. In recent years, stem cell transplantation has attracted more and more attention as a new technique for treating diabetic lower limb ischemic disease, including the diabetic foot. Stem cell therapy aims to stimulate the formation of new blood vessels to increase blood supply and relieve limb ischemia, ultimately promoting wound healing. Moreover, the administration of stem cells based on traditional treatment undoubtedly better exploit the role of stem cells in repairing damage and greatly improves the negative consequences of severe complications of diabetic foot. For example, angioplasty combined with human umbilical cord mesenchymal stem cells (hUC-MSCs) transplantation can improve the blood supply of severe diabetic foot, promote ulcer healing, reduce amputation rate and mortality, and improve the quality of life of patients with advanced diabetic foot (Qin et al., 2016). In addition, there is evidence that the application of myogenic mesenchymal stem cells (MMSCs) can essentially reverse the vascular occlusion of diabetes-related peripheral artery disease (PAD) (Hedhli et al., 2017).

In contrast to this eminent function of stem cells, however, the mechanisms underlying an impaired wound healing process are poorly understood. Therefore, a better understanding of the characteristics of stem cells and the signals that control their behavior is expected to bring new hope to the treatment of the diabetic foot. This review will begin with an overview of the characteristics of the diabetic foot, followed by an overview of stem cells and their therapeutic potential and our current understanding of extended application of stem cell-based therapy in DFUs.

## Characteristics of the Diabetic Foots

Diabetic foot presents a long-term complex interaction of neuropathic, macrovascular, and microvascular diseases in an abnormal metabolic context, accompanied by a decline in healing ability. Diabetes can cause microvascular dysfunction, and as a result, the microvascular supply to the leg nerves may be affected, resulting in abnormal foot movements (motor nerves), paresthesia (sensory nerves), and decreased sweating (autonomic nerves) (Cavanagh et al., 2005; Grennan, 2019). Asymptomatic diabetic peripheral neuropathy is up to 50% (American Diabetes Association, 2016). Even if patients are symptomatic, less than a third of physicians can recognize the manifestations of peripheral neuropathy associated with diabetes (International Diabetes Federation, 2019). Early symptoms (pain and paresthesia) of diabetic peripheral neuropathy are caused by small fibers, while large fiber lesions cause numbness and loss of protective sensation (LOPS) (Forbes and Cooper, 2013; American Diabetes Association, 2016). Due to LOPS, patients are unaware of minor injuries due to external trauma and/or foot deformity (Steed et al., 2006; Vileikyte et al., 2017). Therefore, these ulcers may have enlarged before they are detected, and by the time they are detected, 25–50% of the foot ulcers already have gangrene (Lepäntalo et al., 2011; Grennan, 2019). Due to some of the above possible reasons, diabetic foot ulcers have become the most ordinary complication of diabetes-related complications. Its lifetime incidence is estimated at 19–34% (Armstrong et al., 2017). The incidence of recurrence of foot ulcers within 5 years is up to 65% (Armstrong et al., 2017).

Although diabetic peripheral neuropathy is a microvascular complication, it may adversely affect microvascular function (Stirban, 2014; Barwick et al., 2016). It has been reported that ischemic ulcers may account for only 10% of diabetic foot lesions, and 90% are caused by neuropathy, alone or in association with ischemia (Boulton, 2010). Diabetic foot is ischemic, and the large blood vessels in the legs may also be affected by diabetes (Grennan, 2019). Compared with lower limb ischemia caused by simple arteriosclerosis, the treatment of diabetes combined with lower limb arterial ischemia is more difficult and complicated. Poor artery flow will reduce blood supply to the ulcer areas, ultimately resulting in impaired oxygen delivery and nutritional supply (Steed et al., 2006; Jiang et al., 2012). These effects may slow the healing of the ulcers. Peripheral nerve injury combined with reduced blood flow perfusion (including large and microvascular vessels) increases the likelihood of foot ulceration, infection, and eventual amputation (Lepäntalo et al., 2011; Bus, 2012). Also, high blood sugar can delay wound healing and worsen the infection (van Crevel et al., 2017; Giri et al., 2018; Grennan, 2019).

At the molecular and cellular levels, abnormalities of cytokines produced by inflammatory cells, decreased macrophage infiltration, reduced growth factor, reduced collagen synthesis and impaired neovascularization make wound healing slow or nonhealing. The interaction of various complex factors eventually makes the wound repair process abnormal, from blood hemostasis and coagulation to inflammation, then epithelialization as well as angiogenesis, and finally granulation tissue formation. Therefore, compared with routine wound healing, the diabetic ulcer has prominent edema and bleeding, dermal or epidermal tissue formation is obstructed, granulation tissue formation is significantly reduced, matrix maturation and remodeling are incomplete, and finally, the loose and irregular connective tissue is formed (Lee et al., 2011).

## Overview of Stem Cells and Their Therapeutic Potential

Stem cells have the characteristics of asymmetric replication, the potential of strong self-renewal, and multi-differentiation (Ding et al., 2011). Asymmetric replication describes the unique properties of stem cells: stem cells undergo mitosis to produce progeny (self-renewal) with the same properties as their mothers and progeny (differentiated cells) with more restrictive potential (Lanza and Atala, 2014).

Embryonic stem cells (ESCs) can be obtained from the inner cell mass of the blastocyst early in development (Bogliotti et al., 2018) or prepared *in vitro* by *in vitro* fertilization (Hikabe et al., 2016), or the nuclei of somatic cells can be transplanted into the enucleated oocytes using cloning techniques and isolated from the cells of their inner cell mass when they reach the blastocyst stage of development (Hochedlinger and Jaenisch, 2003). Despite their extraordinary potential, the use of ESCs in the clinical setting remains limited. First, the use of hESCs is highly ethically controversial. Although ESCs can be obtained without destroying the embryos, it is unclear whether this approach necessarily eliminates ethical issues and whether sufficient ESCs can be generated in this method. In addition, ESCs may not be safe; they are inclined to have a vigorous proliferative capacity and lower differentiation maturity, so the introduction of these cells may cause immune rejection and stimulation of tumor formation. Therefore, ESCs are rarely employed for the treatment of DFUs (Jiang et al., 2012).

Adult stem cells are not ethically controversial and can be isolated from several sources. They were initially found in the bone marrow but are currently thought to exist in almost every organ, including fat, umbilical cord, and placenta (Ding et al., 2011). They have multi-directional differentiation potential and can be induced to differentiate into at least three different types of functional cells, even across germ layers, which is a reversal of the traditional understanding that adult stem cells can only differentiate into functional cells of the corresponding germ layer (Lanza and Atala, 2014). Although adult stem cells are less potent, they are still significant for maintaining overall health.

Nerves, which were previously thought to be non-regenerative, have now been found to be regeneratively repairable through stem cell differentiation (Mathot et al., 2019). By injecting stem cells into the patients’ muscles or arteries or applying them directly to the wound locally, stem cells can be localized to the injured tissue by some mechanism and promoted epidermal regeneration by secreting cytokines to promote neovascularization. In addition, coldness and pain caused by nerve ischemia may be relieved (Wang et al., 2019).

Different types of stem cells can improve chronic diabetic wounds by enhancing the expression levels of vascular endothelial growth factor (VEGF), promoting cell proliferation, angiogenesis, and granulation tissue formation in traumatic tissues (Lee et al., 2011; Wan et al., 2013). In addition, inflammation and immune regulation also play an essential role in promoting wound healing (Wetzler et al., 2000; Loretelli et al., 2020). Stem cells have been shown in experiments to successfully cure DFUs, offering new prospects for the development of effective therapies for clinical use. Currently, 40 clinical trials have been found on a search for “diabetic foot ulcers, stem cell” (Clinicaltrials.gov, 2021).

## Molecular Mechanisms to Repairing DFUs

### Repairing Diabetic Foot by Promoting Angiogenesis

Due to ethical constraints, the research of ESCs in wound repair is limited to a certain extent. However, some animal experiments have shown that ESCs have broad prospects in repairing chronic wounds, especially during the early stage of wound repair. Lee et al. (2011) found in the experiment of topical application of ESCs on the full-thickness skin of diabetes-induced rats that the expression levels of fibronectin, VEGF, and epidermal growth factor (EGF) are significantly higher than those of the insulin-treated diabetic group.

**FIGURE 1:**
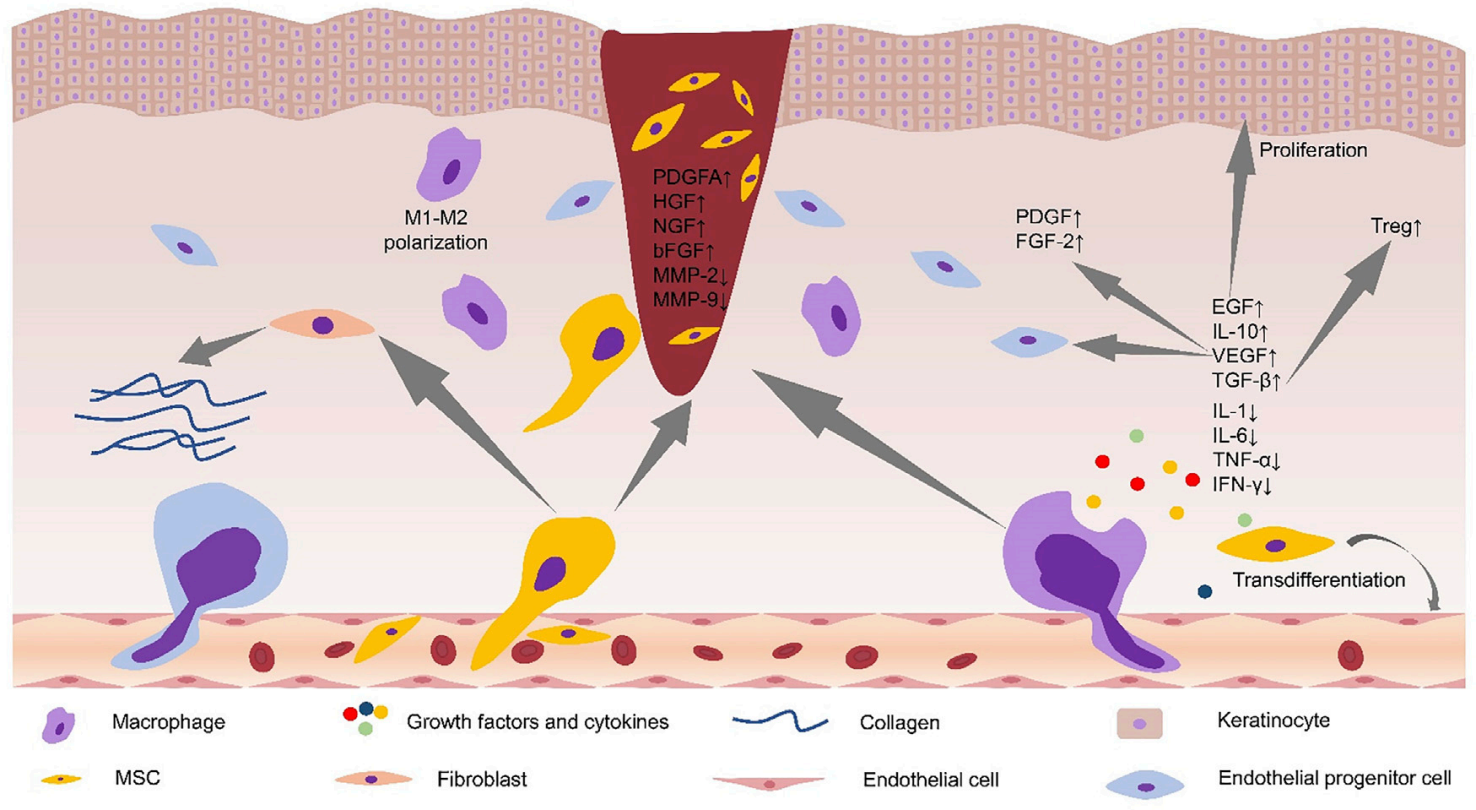
Alterations in the microenvironment of diabetic foot ulcers after stem cell application. Stem cells promoted M2 polarization of macrophages and secretion of anti-inflammatory cytokines. Stem cells increased the levels of PDGFA, HGF, NGF, and bFGF in the wound site, and decreased the levels of MMP-2, MMP-9, and proinflammatory cytokines. Stem cells promoted collagen deposition by acting on fibroblasts. Increased levels of local VEGF can recruit EPCs to the wound site and increase the levels of PDGF and FGF-2. EGF can promote the proliferation of keratinocytes, and TGF-β can regulate local immunity by increasing the levels of Treg cells. MSC, mesenchymal stem cell; PDGFA, platelet-derived growth factor A; HGF, hepatocyte growth factor; NGF, nerve growth factor; bFGF, basic fibroblast growth factor; MMP-2, matrix metaroprotease-2; MMP-9, matrix metaroprotease-9; FGF-2, fibroblast growth factor; EGF, epidermal growth factor; IL-10, interleukin-10; TNF-α, tumor necrosis factor-α; Tregs, regulatory T cells; IFN-γ, interferon-γ; TGF-β, transforming growth factor-β.

Currently, mesenchymal stem cells (MSCs)-based methods have been proposed as prospective therapies for delayed or impaired healing wounds, and numerous studies have shown that MSCs can promote diabetic wound healing (Table 1). Experiments show that intramuscular transplantation works best (Wan et al., 2013). MSCs can systematically mobilize and find home for injured and ischemic tissues, creating a wound microenvironment conducive to wound healing and ultimately promote the wound healing process of diabetic foot (Zhang et al., 2010; Wan et al., 2013; Shi et al., 2016; Shi et al., 2020) (Figure 1). Wound healing is a complex and multifactorial tissue regeneration process involving the interaction between epidermal and dermal cells, as well as the interaction between cells and cytokines. For example, Shi et al. (2020) detected increased levels of VEGF, basic fibroblast growth factor (bFGF), and hepatocyte growth factor (HGF) in DFUs treated with UC-MSCs. HGF, a downstream pathway of PI3K, can upregulate VEGF expression through the c-MET signaling pathway (Matsumura et al., 2013). TNF-α, as an essential component at the wound site, could secret VEGF and HGF by stimulating hMSCs *via* a p38 MAPK-dependent mechanism (Wang et al., 2006). Cytokines and growth factors affect the activity of other cells (including immune cells) and are necessary for communication between various cells (e.g., fibroblasts, keratin-forming cells, immune cells, and endothelial cells) (Matthay et al., 2010). A study concluded that VEGF could locally upregulate platelet-derived growth factor B (PDGF-B) and fibroblast growth factor-2 (FGF-2) in wounds (Galiano et al., 2004) and systematically mobilize and recruit bone marrow-derived cells (including endothelial progenitor cells (EPCs)) into the local microenvironment of wound site to improve wound healing and tissue remodeling (Galiano et al., 2004; Ishida et al., 2019).

**TABLE 1:** Potential role of stem cells in the healing process of DFUs.

References	Cell type	Objective	Mode of administration	Changes in the molecular level	Changes in histology/Clinical manifestation
Lee et al. (2011)	ESCs	STZ-induced T1DM in Sprague-Dawley rats	Topical application	EGF and VEGF↑, fibronectin levels↑	High vascular counts, moderate re-epithelialization, well-formed granulation tissue formation, and capillary vessels
Loretelli et al. (2020)	ESCs	Db/db mice*	Topical application of EXTs	Tregs↑, proliferating Ki-67^+^ cells↑, CD31^+^ endothelial cells↑, CD45^+^ inflammatory cells↓, IFN-γ↓, Th1↓	Re-epithelialization, angiogenesis, and reduced leukocyte infiltration
Wan et al. (2013)	BM-MSCs	STZ-induced T1DM in Wistar rats	Intramuscular transplantation	VEGF↑, proliferating Ki-67^+^ cells↑, CD31^+^ endothelial cells↑	Angiogenesis, cellular proliferation, and granulation tissue formation
Kuo et al. (2011)	BM-MSCs	STZ-induced T1DM in Wistar rats	Subdermal injection	CD45↓, VEGF↑, EGF↑, prolyl 4-hydroxylase↑, Ki-67 expression↑	Proinflammatory reaction↓, wound sizes↓
Dash et al. (2009), Wang et al. (2019)	BM-MSCs	24/4 diabetic patients	Intramuscular transplantation	—	Accelerate the healing process, pain relief, wound size↓
Kato et al. (2014)	BM-MSCs	STZ-induced T1DM in Sprague-Dawley rats	Topical application	Expression levels of MMP-2, EGF, IGF-1, and pFAK↑, the expression level of MMP-9↓	Wound size↓
Uchiyama et al. (2017)	BM-MSCs	Db/db mice; WT mice; and C57BL/6 mice	Intradermal injection	IL-10↑, TNF-α↓, the number of M2 macrophages↑	Promote angiogenesis and accelerate diabetic wound healing
Lv et al. (2017)	BM-MSCs/stem cells from human SHED	STZ-induced T1DM in Sprague-Dawley rats	Topical application	Expression levels of VEGF, eNOS, MMP-2 and MMP9↑, the expression of IL-1β, IL-10, and TNF-1α↓	Inflammatory cells↓, epithelialization↑, well formation of granulation tissue
Zhao et al. (2013)	hUC-MSCs	STZ-induced T1DM in Sprague-Dawley rats	Left femoral artery injection	Cytokeratin 19↑, collagen I and collagen III↑, the ratio of collagen I/III↓	—
Xia et al. (2015)	hUC-MSCs	STZ-induced T1DM in Sprague-Dawley rats	Left femoral artery injection	Serum NGF↑	Numbers of capillary↑
Yue et al. (2020)	hUC-MSCs	STZ-induced T1DM in Sprague-Dawley rats	Subcutaneous injection	Expression levels of PDGFA and HGF↑	Accelerate diabetic wound healing, angiogenesis, and re-epithelialization
Shi et al. (2020)	hUC-MSCs	STZ-induced type 1 diabetes in Sprague-Dawley rats	Transfemoral vein transplantation	Ki-67 + cells↑, VEGF, bFGF, and HGF↑, IL-1ra, IL-10, CINC-1, CINC-2α/β, CINC-3, CNTF, CCL3, CCL5, CCL20, CX3CL1, CXCL7, and LIX↑, IL13↓	Collagen deposition and vascular density↑, closely complete re-epithelialization, fewer infiltrating inflammatory cells, and thick granulation tissue
Li et al. (2013)	hUC-MSCs	15 diabetic patients	Intramuscular injection	The ratios of Treg/Th17 and Treg/Th1 cells↑, VEGF↑, IL-6↓	—
Zhang S et al. (2020)	UCMSCs	STZ-induced C57BL/6J female mice	Subcutaneous injection	IL-10↑, IL-1β, TNF-α, and IL-6↓, the number of M2 macrophages↑	Skin angiogenesis
Shi et al. (2016)	HADSCs	STZ-induced type 1 diabetes in Sprague-Dawley rats	Transfemoral vein transplantation	IL-1ra, IL-2, TNF-α, and CNTF↓, IL-1β, IL-6, IL-13, CCL3, CINC-1, CINC-2α/β, CINC 3, CX3CL1, LECAM-1, and LIX↑, VEGF, bFGF, and TGF-β↑	Epithelialization↑, well formation of granulation tissue
De Gregorio et al. (2020)	HADSCs	Db/db mice*	Systemic administration	Expression of IL-1β and TNF-1α↑	Reducing chronic inflammation of peripheral nerves and improving angiogenesis
Uzun et al. (2020)	HADSCs	20 patients	Dermo-epidermal junction injection	—	Wound size↓
Maharlooei et al. (2011)	HADSCs	STZ-induced type 1 diabetes in Sprague-Dawley rats	Subdermal injection	—	Wound size↓
An et al. (2020)	HADSCs	STZ-induced C57BL/6J male mice	Topical application	Collagen I and collagen III↑, IL-6↓	Skin angiogenesis
Wang et al. (2016)	PMSCs	GK rats†	Subcutaneous injection	TNF-α, IL-6 and IL-1↓, IL-10↑	Angiogenesis, collagen deposition and thick granulation tissue, the infiltration of macrophage↓
Zhao et al. (2020a)	Combined use of ECFCs, HA, and UBC-MSCs	12 subjects	Topical cell injection	—	Re-epithelialization rate↑, wound size↓
Yang et al. (2020a)	Combination product of dermal matrix, timolol (beta-adrenergic antagonist), and BM-MSCs	Db/db mice*	Scaffold’s implantation	CD31 + cells↑, CD45 + cells↓, CCL2 expression level↑, IL-1β, IL-6, CXCL-1, and CXCL-2↓	Anti-inflammatory and pro-angiogenic functions↑

**FIGURE 2:**
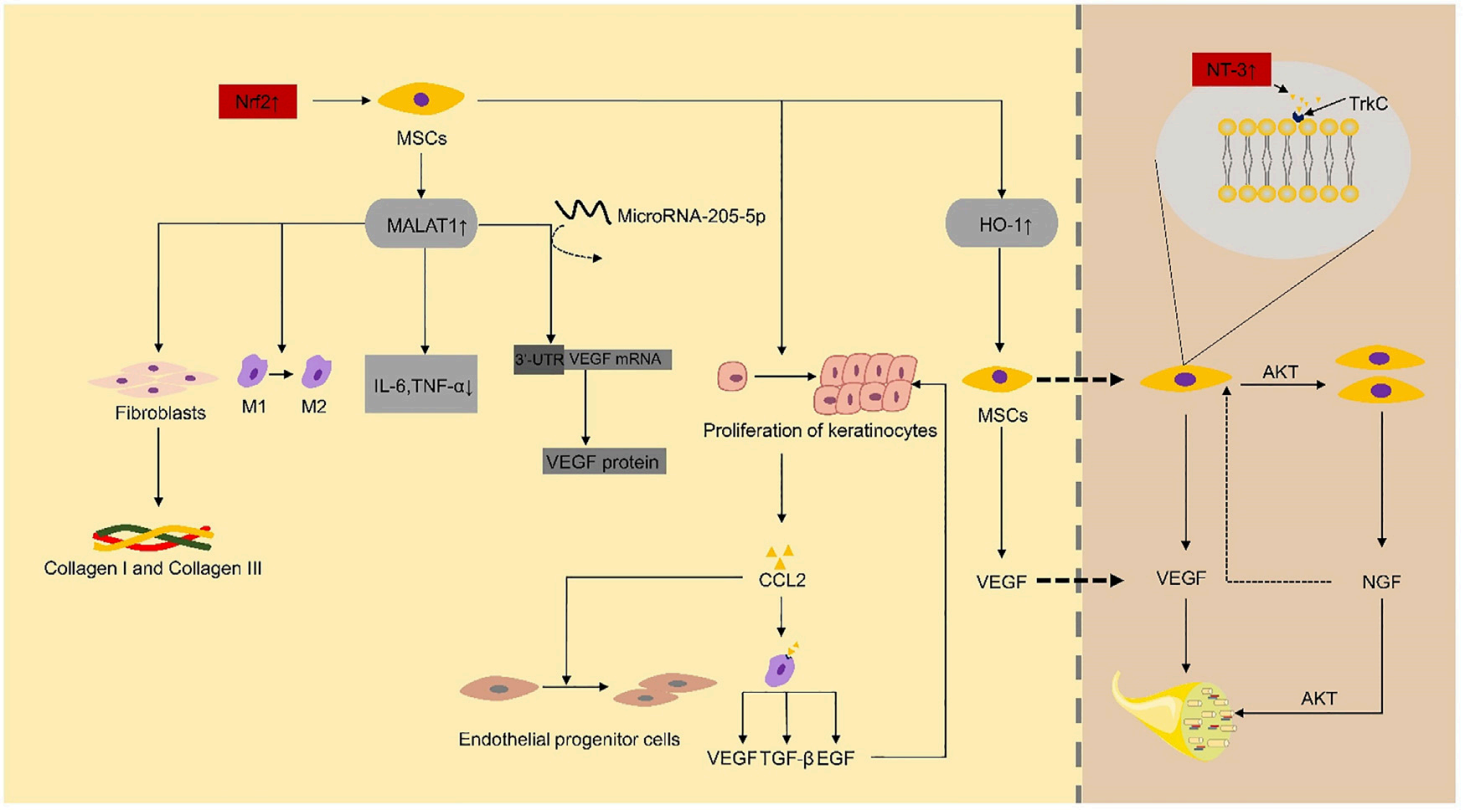
Nrf2 and NT-3 play a critical role as key factors in the repair of diabetic foot ulcers by mesenchymal stem cells. IL-6, interleukin-6; TNF-α, tumor necrosis factor-α; TGF-β, transforming growth factor-β; EGF, epidermal growth factor; VEGF, vascular endothelial growth factor; MSCs, mesenchymal stem cells; NGF, nerve growth factor; Trk, tropomyosin receptor kinase.

Neovascularization, a critical step for the process of wound healing, was consistently depressed by hyperglycemic conditions at the wound sites, as revealed by endothelial cell marker CD31 (Ishida et al., 2019). MSCs can significantly improve diabetic foot ulcers by producing angiogenic factors such as VEGF. Compared with non-diabetic rats, the expression level of VEGF protein in diabetic rats was significantly reduced (Schratzberger et al., 2001). MicroRNA-205-5p is a direct regulator of VEGF protein translation and is expressed in hMSCs (Zhu et al., 2017). Zhu et al. (2019) found that microRNA-205-5p could specifically target 3′-UTR of VEGF mRNA to inhibit the translation of VEGF protein, thus inhibiting wound healing of diabetic foot (Zhu et al., 2017). MALAT1 is a competing endogenous RNA (ceRNA) competing with microRNA-205-5p. The expression of MALAT1 was downregulated in tissue biopsies of DFU patients (Jayasuriya et al., 2020). In the DF model established by immunodeficient NOD/SCID mice, the overexpression of MALAT1 in MSCs significantly downregulated the level of microRNA-205-5p, led to the up-regulation of VEGF production and improved the formation of endothelial cell tubes (Zhu et al., 2019). Deletion of microRNA-205-5p and overexpression of MALAT1 did not substantially change the mRNA level of VEGF but significantly enhanced the protein level of VEGF in MSCs and MALAT1 serves as a post-transcriptional activator to increase the expression of VEGF protein (Zhu et al., 2019). MALAT1 silencing can reduce the expression level of Collagen I and Collagen III in the skin of diabetic mice, thus reduce the deposition of collagen at the wound sites and delay wound healing (Liu et al., 2019). Collagen is secreted by fibroblasts (Vig et al., 2017). These studies indicate that MALAT1 promotes ulcer healing by promoting VEGF secretion or acting on fibroblasts to promote collagen secretion. The level of Nrf2 in the circulation of diabetic patients is lower (Sireesh et al., 2018). In diabetic foot ulcers, Nrf2 can upregulate the expression of MALAT1 through a positive feedback mechanism (Jayasuriya et al., 2020). Exosomes from adipose-derived stem cells (ADSCs) overexpressing Nrf2 improve angiogenesis and proliferation of EPCs, which accelerate cutaneous wound repair in DFUs (Li et al., 2018). These results support a crucial role for Nrf2 in modulating the effects of MSC-based therapies in the unfavorable *in vivo* microenvironment (Figure 2). Furthermore, increasing the local MALAT1 level of wounds can significantly improve wound inflammation and promote wound healing. For example, MALAT1-overexpressed MSCs can promote M2 macrophage polarization (Li et al., 2017) and decrease the expression of proinflammatory cytokines IL-6 and TNF-α (Jayasuriya et al., 2020).

Nrf2-dependent genes, such as heme oxygenase-1 (HO-1), play a vital role in the regulation of angiogenesis and the increased expression of angiogenic factors (Loboda et al., 2016). Hou et al. (2013) reported that the overexpression of HO-1 in BMSCs in STZ-induced diabetic mice could promote the proliferation of BMSCs and enhance the secretion of VEGF by BMSCs through the Akt signal pathway, which eventually promotes wound ulcers repair significantly.

MFG-E8 is expressed perivascularly and intravascularly, and it is essential for VEGF-induced AKT phosphorylation (Silvestre et al., 2005). VEGF and PDGF, as crucial growth factors for the induction of angiogenesis, require MFG-E8-mediated signaling (Silvestre et al., 2005; Uchiyama et al., 2014). A study confirmed that BM-MSCs could promote angiogenesis by overexpressing MFG-E8 (Uchiyama et al., 2017). Although this study also shows that MFG-E8 can promote M2 macrophage infiltration and inhibit proinflammatory cytokines, thus promoting diabetic wound healing, the underlying mechanism of MFG-E8 regulating inflammation is not clear.

PI3K/Akt signal pathway is essential for angiogenesis (Karar and Maity, 2011). Nerve growth factor (NGF), which binds to its specific receptor TrkA (Storkebaum and Carmeliet, 2011), can promote the proliferation of endothelial cells in blood vessels and stimulates MSC to produce VEGF (Shen et al., 2013) and nitric oxide synthase (NOS) through PI3K/Akt pathway to promote angiogenesis (Emanueli et al., 2002; Emanueli et al., 2003). MSCs expressed NT-3 specific receptor type 3 neurotrophin tyrosine kinase receptor (TrkC) (Storkebaum and Carmeliet, 2011). NT-3-activated hMSC can promote foot wound healing in diabetic mice. Shen et al. (2013) reported that NT-3 upregulates the proliferation of hMSC through the Akt pathway. NT-3 can also upregulate the secretion of VEGF and NGF proteins in MSCs, and these growth factors can enhance the activity of vascular endothelial cells and promote angiogenesis through Akt pathway (Shen et al., 2013).

The c-jun expression of MSCs decreased gradually in the process of subcultivation (Yue et al., 2020). At the diabetic wound sites, the expression of c-jun also decreased (Tombulturk et al., 2019). Yue et al. (2020) found that local subcutaneous injection of hUC-MSCs overexpressing c-jun at the diabetic wound sites accelerated angiogenesis and re-epithelialization by increasing the levels of PDGFA and HGF. PDGFs are the major mitogens for several cells types of mesenchymal origin, such as smooth muscle cells and fibroblasts (Fredriksson et al., 2004). Activator protein-1 (AP-1) is one of the main downstream effectors of MAPKs (Kajanne et al., 2007), and c-Jun, the main component of the AP-1, has crucial functions in cellular proliferation (Davies et al., 2013). The c-jun expression of MSCs plays a vital role in promoting diabetic foot wound repair. MSCs overexpressing c-jun may promote wound repair by downregulating the expression of matrix metalloproteinase-2 (MMP-2) and matrix metalloproteinase-9 (MMP-9). MMP-2 and MMP-9 are overexpressed in diabetic chronic ulcers (Wysocki et al., 1993). AP-1 has shown to be the main regulator of MMP-2 and MMP-9 transcription under various conditions, as well as in diabetic wounds (Tombulturk et al., 2019). Although the local application of bone marrow MSCs resulted in increased levels of MMP-2 and MMP-9 expression in diabetic wounds compared to the control group, they showed a decreasing trend (Lv et al., 2017). In the process of wound healing, MMP-2 plays an essential role in prolonged matrix remodeling and angiogenesis (Tombulturk et al., 2019), and MMP-9 is thought to be involved in granulation tissue remodeling and keratinocyte migration (Salo et al., 1994). In addition, there has been evidence that down-regulation of MMP-2 and MMP-9 by c-jun can promote wound healing in diabetic mice (Tombulturk et al., 2019), and the overexpression of MMP-2 and MMP-9 can impair wound healing through the degradation of skin extracellular matrix (ECM) (Krishnan et al., 2018).

### Repairing Diabetic Foot Through Anti-Inflammatory and Immunomodulation

Long-term inflammation can lead to a hypoxic environment and abnormal produc tion of angiogenic signals. The anti-inflammatory and immunomodulatory effects of implanted stem cells have been suggested as a potential mechanism for the repairing effects of stem cell-based therapies. Inflammation has a regulatory effect on immune function. Low-grade inflammation is beneficial to wound repair, which helps to remove invading pathogens, promotes the repair of damaged tissues, and maintains homeostasis. Continuous and excessive inflammation leads to delayed or nonhealing of diabetic foot wound ulcers (Eming et al., 2007). Excessive inflammation and immune suppression often coexist. The excessive immune response can cause severe systemic inflammation or allergic diseases, while an extremely low immune response can easily induce severe or frequent infection. Therefore, the balance between proinflammatory and anti-inflammatory during varying repair stages is essential to achieving tissue homeostasis following tissue injury. Loretelli et al. (2020) found that the topical application of embryonic stem cell extracts (EXTs) in diabetic foot mice can promote wound healing of diabetic foot, which is accompanied by a decrease in CD45^+^ inflammatory cells and interferon-γ (IFN-γ), while an increase in regulatory T cells (Tregs), proliferating Ki-67+ cells, and the endothelial cell marker CD31. This finding indicates that the promotion of diabetic wound healing by ESCs is related to immune regulation. It is well known that stem cells have anti-inflammatory and immunomodulatory properties, so it is of great significance to explore the relationship between the immunomodulatory properties of ESCs and wound healing.

Delayed increase of macrophages and prolonged inflammatory stage can be found in diabetic mice. The local proinflammatory response in the MSC group was significantly reduced comparing with the control group, and the expression of CD45 was inhibited, proving that MSCs play an anti-inflammatory effect in repairing diabetic foot ulcers (Kuo et al., 2011; Yang et al., 2020a). Furthermore, the abnormal expression of chemokines may be the underlying cause of delayed wound healing in diabetic mice (Wetzler et al., 2000). A study has shown that the concentration of CCL2 in diabetic wounds is reduced, and an enhanced expression of CCL2 can reverse the impaired skin wound healing in diabetic mice by mediating neovascularization and normalization of collagen accumulation (Ishida et al., 2019). Macrophages were the main source of VEGF and TGF-β in the wound, and diabetes can delay the increase of macrophages at the injured site. More than a few studies have demonstrated MSCs can enhance the levels of VEGF and TGF-β at the diabetic wound sites (Kuo et al., 2011; Wan et al., 2013; Yang et al., 2020b). Yang et al. (2020a) demonstrated that the expression of CCL2 was considerably upregulated after topical application of MSCs, and angiogenesis was markedly improved during wound healing. Macrophages express CCL2 receptors (CCR2). Therefore, it can be speculated that CCL2 plays an important role in promoting wound repair by reversing the reduced macrophage infiltration and enhance the levels of VEGF and TGF-β. TGF-β can directly promote the proliferation of Treg cells and regulate macrophages (Batlle and Massagué, 2019). Nrf2 directly regulates the secretion of CCL2 by epidermal keratinocytes (Villarreal-Ponce et al., 2020). Kato et al. (2014) have shown that BM-MSCs can improve wound healing of plantar skin ulcers in diabetic rats by enhancing the function of keratinocytes. Therefore, we can speculate that MSCs may increase the secretion of CCL2 by promoting the functional recovery of keratinocytes, which can reverse the decrease of macrophage infiltration and ultimately promote ulcer repair. CCL2 can also regulate the production of EGF in macrophages at the site of injury, and EGF can induce the proliferation of keratinocytes (Villarreal-Ponce et al., 2020).

Hyperglycemic conditions have also been shown to reduce the number of EPCs and impair their function and recruitment to the injured site (Tepper et al., 2002; Ishida et al., 2019). The increased levels of CCL2 expression can further enhance the accumulation of EPCs in the wound site of diabetic mice and ultimately accelerate the formation of new blood vessels (Ishida et al., 2019).

Diabetic chronic nonhealing wounds are characterized by the increase of proinflammatory cytokines, including IL-1, IFN-γ, TNF- α, and IL-6, which are mainly produced by activated macrophages and play an important role in the regulation of immune cells (Zubair and Ahmad, 2019). It is suggested that the decrease and activation of macrophages may result in the damage of diabetic wound healing (Maruyama et al., 2007). Topical application of MSCs may modulate the inflammatory response by secreting the anti-inflammatory cytokine IL-10. Wang et al. (2016) found that combined treatment with PMSCs and IL-10 antibodies significantly delayed diabetic wound healing compared to treatment with PMSCs alone. Furthermore, topical application of PMSCs significantly reduced local levels of the proinflammatory cytokines IL-1, TNF-α, and IL-6. The wound inflammation reaction time was significantly prolonged in db/db mice, and the strong increase of proinflammatory cytokines (e.g., IL-1β and TNF-α) could still be detected in the later stage of the wound repair (Wetzler et al., 2000). The presence of PMSCs was found to strongly inhibit LPS-induced activation of NF-κB in dermal fibroblasts. Since NF-κB plays a central role in regulating the transcription of the proinflammatory cytokines IL-1, TNF-α, and IL-6, it is hypothesized that PMSCs may regulate the inflammatory response in diabetic foot wound healing by targeting NF-κB (Wang et al., 2016). In addition, the two proinflammatory cytokines, IL-1β and IL-6, were significantly reduced in wounds of db/db mice treated with the MSC combination regimen (Yang et al., 2020a), supporting its immunomodulatory function. In conclusion, these findings indicate that MSCs promote cutaneous wound healing, at least in part, by inhibiting the secretion of proinflammatory cytokines and by increasing the production of anti-inflammatory cytokines at local wound sites.

Diabetic wounds are characterized by disturbance of macrophage phagocytosis, delayed macrophage infiltration, and inflammatory response disorder. Proinflammatory cytokines (IL-6, TNF-α, IFN-γ) are known to induce M1 macrophages (Gu et al., 2013). M2 macrophage is characterized by the production of TGF-β and IL-10 (Zhang S et al., 2020), which can be induced by anti-inflammatory cytokines, including IL-4, IL-10, and TGF-β (Gu et al., 2013). M2 macrophages have been shown to promote angiogenesis, improve nerve damage, and inhibit inflammation. The microenvironment of wound inflammation determines the phenotype of macrophages. Topical application of stem cells to wounds can promote the conversion of macrophages to an anti-inflammatory phenotype (M2) (Yang et al., 2020a). Depletion of Treg leads to impaired healing, characterized by impaired vascular maturation and delayed re-epithelialization (Haertel et al., 2018). A study has shown that intramuscular injection of hUC-MSCs into patients with diabetic foot can significantly increase the ratios of Treg/Th17 and Treg/Th1 cells (Li et al., 2013). And then, Treg cells induce M2 macrophage differentiation through TGF-β and IL-10 pathways (Liu et al., 2011). Furthermore, M2 macrophages can improve the local inflammatory microenvironment of wounds and thus promote angiogenesis. It is reported that umbilical cord-matrix stem cells (UCMSCs) polarized macrophages can promote high glucose-induced functional recovery of human umbilical vein endothelial cells (Zhang S et al., 2020).

### Repairing Diabetic Foot by Improving Nerve Blood Supply and Reducing Chronic Nerve Inflam Mation

Peripheral neuropathy and microangiopathy of the diabetic foot often develop and aggravate at the same time. Therefore, the repair of nerves may also be one of the possible mechanisms for MSCs to repair diabetic foot ulcers. In fact, the number of blood vessels and nerve blood flow reduced significantly in streptozotocin (STZ)-induced diabetic rats, and intramuscular gene transfer of naked DNA encoding angiogenic growth factors VEGF-1 or VEGF-2 actually led to the resolution of diabetic neuropathy (Schratzberger et al., 2001). Since VEGF improves the blood vessels and blood flow in the nerves in the model, the effect of VEGF in promoting vascular perfusion seems to be related to the improvement of neuropathic diabetic foot. Notably, NGF and NT-3 can reverse diabetic neuropathy (Casellini and Vinik, 2007) (Figure 2). Therefore, we can speculate that NGF and NT-3 can improve neuropathic diabetic foot by acting on VEGF directly or indirectly. Human UC-MSCs improved the neurodegeneration of the femoral nerve in DFU rats and showed an increase in serum NGF and angiogenesis, which further confirmed this view (Xia et al., 2015). In addition, ADSCs can reverse the degeneration of large nerve fibers, promote neuronal axonal regeneration, and improve peripheral nerve perfusion (De Gregorio et al., 2020).

Chronic inflammation of the peripheral nerve directly leads to chronic pain and neurodegeneration (Vincent et al., 2011). De Gregorio et al. (2020) demonstrated that adipose-derived stem cells (ADSCs) can reduce chronic inflammation of the sciatic nerve by upregulating anti-inflammatory cytokines IL-1β and TNF-α. Dash et al. (2009), Wang et al. (2019) reported that patients with DFUs had a significant improvement in painless walking distance after using BM-MSCs.

### Other Mechanisms: Transdifferentiation Into Epithelial Cells, Collagen Deposition Etc

In addition to promoting angiogenesis through paracrine, hUC-MSCs can directly transdifferentiate into epithelial cells and endothelial cells, which play a role in promoting the repair of diabetic foot ulcers (Shi et al., 2020). CD31^+^ cells were presumed to identify transdifferentiated endothelial cells. Consistent with the study that ADSCs can also differentiate into endothelial cells and promote angiogenesis (An et al., 2020), it is suggested that hMSCs have similar characteristics, although they may be through different signal pathways, for example, MAPK/ERK signaling pathway mediates VEGF-induced differentiation of BM-MSCs to endothelial cells (Xu et al., 2008), and PI3K signaling pathway mediates VEGF-induced differentiation of ADSCs to endothelial cells (Cao et al., 2005). However, it is not clear through which signal pathway mediates the differentiation of hUC-MSCs to endothelial cells.

MSCs can improve collagen synthesis and promote collagen deposition in diabetic foot wounds (Wang et al., 2016; Shi et al., 2020). Collagen is mainly expressed and distributed in the dermis of the skin, secreted by fibroblasts, and is closely related to the repair of damaged skin (Vig et al., 2017). Fibroblasts isolated from diabetic foot wounds show lower proliferative potential and reduced production of growth factors (Robson, 2003). ADSCs from diabetic foot wounds can be directly transformed into fibroblasts, which significantly promote the expression of collagen I and collagen III (An et al., 2020). Jung et al. (2018) healthy fibroblasts and human umbilical cord blood-derived mesenchymal stem cells (hUCB-MSCs) were used separately to co-culture with diabetic fibroblasts to study their effects on cell proliferation and collagen synthesis. The results showed that hUBC-MSCs themselves did not secrete more ECM components than fibroblasts, but hUBC-MSCs stimulated the synthesis of ECM (including collagen) in diabetic fibroblasts much more than in fibroblasts. Therefore, MSCs may promote granulation tissue formation in diabetic foot wounds by promoting fibroblast proliferation and functional recovery, thereby prompting fibroblasts to secrete more ECM and growth factors, and ultimately promoting wound healing.

In fact, the process of stem cell repair of diabetic foot is not a single factor acting independently; various mechanisms are interconnected and interact with each other to ultimately promote damage repair. For example, the M2 polarization of macrophages accelerates peripheral nerve repair (Mokarram et al., 2012; Pacific et al., 2020).

## Extended Application of Stem Cell-Based Therapy in DFUs

**FIGURE 3:**
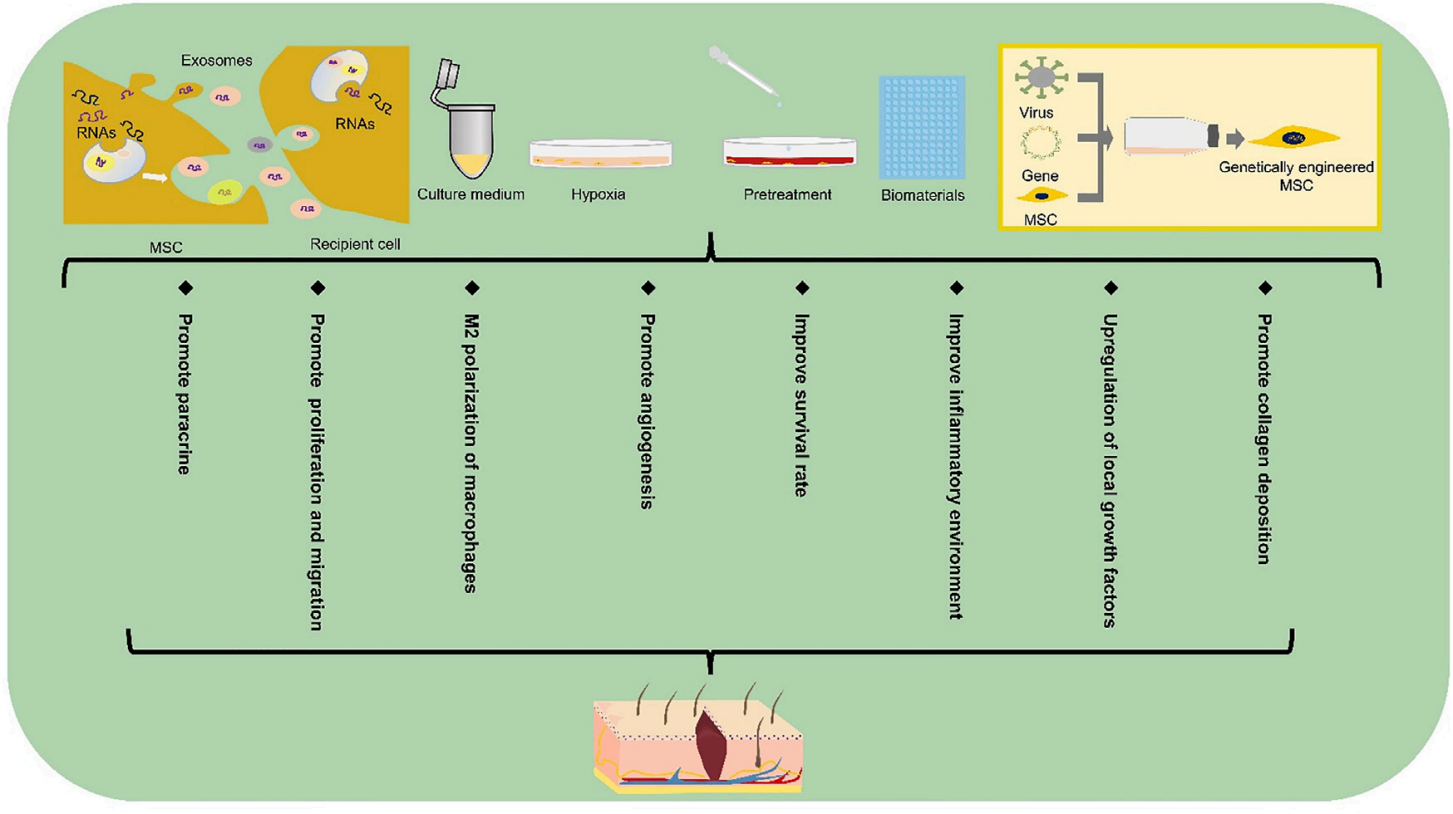
Extended application of stem cell-based therapy in DFUs. Extended applications for stem cell repair of the diabetic foot include exosomes, gene therapy, biomaterials, culture media, pretreatment and hypoxic preconditioning. MSC, mesenchymal stem cell.

The decrease in stem cell survival rate caused by late glycosylation end products in a hyperglycemic environment severely diminishes the efficiency of stem cell repair (Wang et al., 2015). If the repair efficiency is improved by increasing the number of locally applied stem cells, it may increase tumorigenicity and other adverse consequences (Mathew et al., 2016). Moreover, after multiple passages of cells *in vitro*, the potential of multi-directional differentiation and paracrine ability may also decrease. Therefore, there is an urgent need for a method to improve the survival rate and paracrine ability of stem cells. Researchers have begun to develop stem cell-based derived wound repair therapies, which aims to improve the vitality and repair efficiency of stem cells at the wound site. Here, we talk about the extended applications of stem cells (Figure 3).

### Biomaterials

Typical chronic nonhealing wounds show reduced levels of growth factors. Therefore, the wound microenvironment is particularly crucial in wound healing, especially under certain health conditions. Stem cells play a crucial role in regulating the local microenvironment of diabetic foot ulcers. More importantly, these roles have been observed in clinically chronic injury-related diseases, including DFUs (Li et al., 2013). However, the hyperglycemic inflammatory microenvironment may reduce the biological activity of stem cells as well as accelerate the degradation of their secreted cytokines and growth factors. Therefore, a more effective approach to suppress the negative effects of the microenvironment on stem cells appears essential. In recent years, considerable efforts have been contributed to better enhance the repair efficiency and vitality of stem cells and improve the inflammatory microenvironment by using biomaterials as the carrier of stem cell application. Hydrogel has been shown to alleviate the inflammatory environment of the wound, thus promoting the secretion of growth factors from MSCs (Bai et al., 2020) and improving the innervation of diabetic foot (da Silva et al., 2017). Furthermore, Zhao et al. (2020a) managed to improve wound healing by applying hyaluronic acid hydrogel combined with UC-MSCs and endothelial colony-forming cells (ECFCs), which had a significantly positive effect on human refractory diabetic chronic wounds.

ECM-based biomaterials (such as cell sheets and decellularized cell sheet grafts) greatly facilitate stem cell implantation due to their low immunogenicity and resistance to the body’s adverse microenvironment (Capella-Monsonís et al., 2020). Tissue-engineered cell sheets are composed of ECM and cells. It uses temperature-sensitive culture systems or enzyme-sensitive culture systems to naturally desorb cells from the substrate surface and eventually obtain a cell sheet layer structure containing ECM (Zhou et al., 2020). The cell sheet technology can avoid immune rejection caused by exogenous foreign substances such as scaffold materials and maintain the three-dimensional structure of the ECM (Guo et al., 2021). Stem cells, as potential cells with strong regenerative capacity, are the best choice for tissue engineering cell sheets to play a role in damage repair. Adipose stem cell sheets have been shown to accelerate diabetic ulcer healing by upregulating local growth factors (Kato et al., 2015). Although there are no current experiments to validate the survival rate of MSCs at the diabetic foot ulcer sites after cell sheet transplantation, experiments by Guo et al. (2021) demonstrated higher retention and survival rate of stem cell sheets transplanted into the infarcted myocardium than cell suspensions. This provides new hope for improving the survival rate of stem cells from diabetic wound sites. Furthermore, cell sheet-based 3D printing technology makes it possible to form specific shapes to match the structure of the administered tissue or organ while maintaining the structural integrity of the ECM (Bakirci et al., 2017).

Other biomaterials such as peptide-modified biomaterials (Gallagher et al., 2020), cytokine-modified biomaterials (García et al., 2019), injectable biomaterials (Han et al., 2020), and programmable microcapsules (Mao et al., 2019) have also improved the survival rate of cells at the transplantation site to varying degrees, which greatly facilitates MSCs to fully exploit their role in repairing the damage.

### Culture Medium and Exosomes

When stem cells are applied locally to a wound, some of the cells may die in the short term due to hypoxia caused by the local high glycemic environment. However, most experiments have demonstrated that stem cells reach the level of healing injury, can these dead stem cells release a series of bioactive substances to repair the damage after lysis? Moreover, if the bioactive substances released by the dead cells can repair the damage, can there be other alternative methods to replace the direct application of stem cells? It is worth thinking about. Some of the bioactive substances produced by MSCs through paracrine secretion exist in the culture medium used in the culture process of MSC *in vitro*, and these bioactive substances play a crucial role in promoting damage repair. Studies have been conducted to demonstrate that the application of MSC culture medium for the treatment of diabetic foot can achieve the same results as the application of MSCs (Zhang S. et al., 2020). Therefore, the application of MSC conditioned media also brings new hope for patients with diabetic foot ulcers.

Although the application of stem cells has entered the clinical stage, their safety issues still need extensive attention. The weak immunogenicity of stem cells has led to the widespread use of stem cells from different sources in injury repair. However, because of their weak immunogenicity, the application of allogeneic stem cells greatly evades the surveillance of the body’s own immune system and therefore increases the risk of tumorigenicity. In addition, MSCs are highly susceptible to exogenous contamination during their *in vitro* preparation due to inconsistent preparation specifications, which can be extremely harmful to the human body. Most of the therapeutic effects of MSCs are mediated through paracrine secretion, with the most attention being paid to the release of small molecules, exosomes, by stem cells through exocytosis. Exosomes contain a variety of molecules, including proteins, mRNAs, and microRNAs (miRNAs), which can act as novel intercellular communication tools to regulate signaling between target cells. For example, exosome-regulated fibroblasts receive exosome-targeted signals in the form of internalized exosomes and actively regulate collagen production including promotion of type I and type III collagen production during the pre-wound healing phase and inhibition of collagen formation during the late wound healing phase to reduce scar formation (Dalirfardouei et al., 2019). Currently, MSC exosomes have shown promising results in diabetic wounds. For example, exosomes released from menstrual blood-derived mesenchymal stem cells (MenSCs) can improve diabetic foot wound healing by promoting macrophage M2 polarization and angiogenesis (Dalirfardouei et al., 2019). Huang et al. (2021) found that MSC-derived exosome miRNA-21-5p could promote angiogenesis in diabetic ulcers. Even more innovative, researchers have used UCMSCs-derived exosomes combined with hydrogels to promote diabetic foot wound repair (Yang et al., 2020b).

### Gene Therapy

Gene therapy aims to achieve the goal of targeted treatment of diseases by enhancing cell performance, continuously producing functional proteins, and inducing the expression of functional genes in tissues (Shomali et al., 2020). For example, PDGF-B-transfected MSCs can promote traumatic collagen deposition and angiogenesis (Li et al., 2007). A variety of exogenous target genes can be integrated into MSCs genomic DNA and expressed for a long period of time, which is why MSCs have been successfully used as an ideal target cell for the treatment of diseases such as hematological disorders, lung injury, and bone defects. In addition, transfected MSCs have been shown to improve the low survival rate of transplanted cells in diabetic wound sites (Khalid et al., 2019). Researchers have already transfected BM-MSCs with adenovirus-vectored VEGF genes, which significantly promoted the repair of diabetic foot wounds in rats (Cai et al., 2014). Compared with untransfected MSCs, IL-7-transfected BM-MSCs were able to enhance cell-cell connections in diabetic foot trauma as well as promote angiogenesis through induction of angiogenic genes (Khalid et al., 2019). Because of the ability to stably express target genes transduced into cells, exploring specific target genes to enhance the efficacy of MSCs in repairing diabetic foot injury offers a promising future for regenerative medicine.

### Small Molecule Compounds and Drug Pretreatment

The current pretreatment methods mainly include drug pretreatment and small molecular compound pretreatment. As pretreatment can effectively improve the survival rate and paracrine ability of implanted stem cells in hyperglycemic microenvironment, it has been concerned by more and more scientific researchers. The researchers found that UCMSCs pretreated with small molecular compound 3,3′-diindolylmethane could enhance their proliferation and paracrine ability (Shi et al., 2017). In addition, pretreatment with the drug salidroside was able to increase the survival rate of MSCs and improve the migration ability of MSCs in a hyperglycemic microenvironment (Ariyanti et al., 2019). These studies support the positive effects of small molecules or drug pretreatment on stem cells. However, the bioactivity and stability of these small molecules or drugs *in vitro* and *in vivo* are important factors to be considered before applying them in pretreatment, and not all substances with proven *in vivo* or *in vitro* effects such as antioxidant and anti-apoptotic effects can be applied to enhance the repair efficiency of MSCs. For example, curcumin-pretreated MSCs have been shown to be able to withstand oxidative stress (Liu et al., 2015). However, due to the structural instability of curcumin, some scholars have questioned whether it really has some therapeutic efficacy *in vivo* (Nelson et al., 2017).

### Hypoxic Preconditioning

Hypoxic preconditioning can improve the survival rate and wound repair performance of MSC in ischemic and hypoxic environment, mainly through up-regulation of genes related to cell growth, metabolism and stress response pathway (Peck et al., 2019), up-regulation of epithelial regeneration-related genes and angiogenesis-related genes (Zhang X. R. et al., 2020), and restoration of paracrine function damaged in high glucose environment (Xu et al., 2020). The results of hypoxic preconditioning of MSCs by Peck et al. (2019) demonstrated the positive effects of preconditioned MSCs in avascular tissues such as intervertebral discs and cartilage. DFUs also face similar hypoxia as in the intervertebral discs or cartilage due to poor angiogenesis in the acquired wound site. Beegle et al. (2015) found that hypoxia-preconditioned MSCs injected intramuscularly into mice showed reduced glucose consumption and high survival rate. The hypoxic environment is simulated *in vitro* by increasing the CO_2_ concentration (5%) or decreasing the oxygen concentration (1–2%) (Meng et al., 2018; Wang et al., 2018; Zhao et al., 2020b). Hypoxia inducible factor-1α (HIF-1α) is the main regulator involved in hypoxic preconditioning (Peck et al., 2019). HIF-1α expression tended to increase in stem cells as oxygen concentration decreased (Zhao et al., 2020b). Under hypoxic preconditioning conditions, lincRNA-p21 enhanced the repair efficiency of MSCs through the HIF-1α signaling pathway (Meng et al., 2018). HIF-1α positively regulates the expression of its downstream signaling genes such as VEGF, SDF-1a, FGF2, CXCL12, and GRP78 under hypoxic conditions (Lee et al., 2017; Zhao et al., 2020b; Xu et al., 2020). The expression and activity of HIF are suppressed in the hyperglycemic environment of diabetes (Catrina and Zheng, 2021), therefore, hypoxic preconditioning of MSCs appears to be essential in diabetic foot ulcers as a means of enhancing the expression of HIF, the upstream factors regulating damage-repair-related genes. After hypoxia treatment, the expression of cMET in MSCs increased and showed hyperphosphorylation in the early stage under the stimulation of HGF, and even in the later stage, cMET phosphorylation levels were comparable to those in normoxic cultured MSCs (Lee et al., 2017). In addition, hypoxic preconditioning has been demonstrated to improve the tolerance of cells in the corresponding hypoxic environment (Wang et al., 2018).

## Concluding Remarks

Stem cell-based therapies are promising in the field of regenerative medicine, and their mechanisms include promoting angiogenesis, ameliorating neuroischemia and inflammation, and promoting collagen deposition. However, little is known about their specific molecular mechanisms and biological properties. Although the application of stem cells has entered the clinical stage (Table 2), the discussion about their safety remains an inescapable part of the research. MSCs promote wound healing in a dose-dependent manner (O'Loughlin et al., 2013), but at the same time, promote tumor growth in a dose-dependent manner (Djouad et al., 2003). Currently, there are no clear standards regarding the dose of MSC application. Therefore, although stem cells show a bright future in regenerative medicine, there remain numerous challenges for researchers to overcome. In order to maximize the performance of stem cells in repairing skin damage, it is necessary to compare the efficacy and safety of different applications such as local application and systemic injection. Presently, the main ways of applying MSCs in wound sites are local injection, intramuscular injection, intravenous injection, arterial injection, and stent implantation. Systemic infusion requires a larger volume of stem cells to ensure that a sufficient number of stem cells are returned to the target tissue. Although stem cells can be isolated from many tissues, such as bone marrow, umbilical cord, fat, the number of isolated and purified stem cells is not considerable. The homing ability of stem cells after multiple passages was significantly weaker than that of freshly isolated stem cells (Rombouts and Ploemacher, 2003). This leads to an irreconcilable contradiction between the quantity and efficiency of stem cells. In addition, stem cells are blocked in the lungs after intravenous injection, which affects stem cells homing to inflammation or wound sites (Becker and Riet, 2016). Therefore, there remain many issues to be solved in the clinical application of stem cells.

**TABLE 2:** Documented efficacy and limitations of extended application of stem cell-based therapies and brief summary of major trials for each modality.

Extended application of stem cell-based therapies	Efficacy	Limitations	Completed or ongoing trials	Interventions
Biomaterials	Enhance the repair efficiency and vitality of stem cells, improve the inflammatory microenvironment, avoid immune rejection, and improve the retention and survival rate of stem cells	—	Safety, tolerability and efficacy of CYP-006TK in adults with diabetic foot ulcers	Combination product: CYP-006TK
			Clinical study to evaluate efficacy and safety of ALLO-ASC-DFU in patients with diabetic wagner grade 2 foot ulcers	Biological: ALLO-ASC-DFU|procedure: vehicle sheet
			Clinical Study of ALLO-ASC-SHEET in Subjects with Diabetic Wagner Grade II Foot Ulcers	Biological: ALLO-ASC-SHEET
			Treatment of Chronic Wounds in Diabetic Foot Syndrome with Allogeneic Adipose Derived Mesenchymal Stem Cells	Biological: application of allogeneic ADSC stem cells in fibrin gel|Procedure: standard care in diabetic foot ulcer
			Clinical Study of ALLO-ASC-SHEET in Subjects with Diabetic Foot Ulcers	Biological: ALLO-ASC-DFU|procedure: hydrogel sheet (vehicle control)
Culture medium and Exosomes	Modulation of signal transduction between target cells by small molecules	Increases the risk of tumorigenicity and exogenous contamination	—	
Gene therapy	Enhance cell performance and improve the survival rate of stem cells	—	Safety and Efficacy study of neovasculgen (Pl-VEGF165) gene therapy in patients with diabetic foot	Neovasculgen
Small molecule compounds and drug pretreatment	Improve the survival rate, proliferation, migration and paracrine ability of stem cells	Poor bioactivity and stability of small molecules or drugs	—	
Hypoxic preconditioning	Improve the tolerance of cells in the hypoxic environment	—	—	

Reference: https://ClinicalTrials.gov/.
